# Neurofilament light chain for assessment of brain injury in four porcine cardiac arrest models

**DOI:** 10.1016/j.resplu.2026.101466

**Published:** 2026-08-25

**Authors:** Olof Persson, Attila Frigyesi, Anna Valerianova, Mikuláš Mlček, Renaud Tissier, Fanny Lidouren, Matthias Kohlhauer, Giuseppe Ristagno, Aurora Magliocca, Francesca Fumagalli, Kaj Blennow, Henrik Zetterberg, Mohamad Hakam Tiba, Cindy H. Hsu, Thomas H. Sanderson, Robert Neumar, Hans Friberg

**Affiliations:** a Department of Clinical Sciences, Anesthesiology and Intensive Care, Lund University, Lund, Sweden; b Department of Intensive and Perioperative Care, Skåne University Hospital, Lund, Sweden; c Institute of Physiology, First Faculty of Medicine, Charles University, Prague, Czech Republic; d Ecole nationale vétérinaire d’Alfort, IMRB, Inserm U955, After ROSC Network, Maisons-Alfort, France; e Department of Pathophysiology and Transplantation, University of Milan, Milan, Italy; f Department of Cardiovascular Medicine, Istituto di Ricerche Farmacologiche Mario Negri IRCCS, Milan, Italy; g Department of Psychiatry and Neurochemistry, Institute of Neuroscience and Physiology, the Sahlgrenska Academy at the University of Gothenburg, Mölndal, Sweden; h Clinical Neurochemistry Laboratory, Sahlgrenska University Hospital, Mölndal, Sweden; i Department of Pathology and Laboratory Medicine, University of Wisconsin School of Medicine and Public Health, Madison, WI, USA; j Wisconsin Alzheimer’s Disease Research Center, University of Wisconsin School of Medicine and Public Health, University of Wisconsin-Madison, Madison, WI, USA; k Department of Neurodegenerative Disease, UCL Institute of Neurology, Queen Square, London, UK; l UK Dementia Research Institute at UCL, London, UK; m Centre for Brain Research, Indian Institute of Science, Bangalore, India; n Department of Emergency Medicine, University of Michigan, Ann Arbor, MI, USA; o Department of Intensive and Perioperative Care, Skåne University Hospital, Malmö, Sweden

**Keywords:** Cardiac arrest, Swine, Porcine, Neurofilament light chain (NfL), Functional outcome

## Abstract

**Background:**

Anoxic brain injury is the leading cause of death following successful resuscitation after out-of-hospital cardiac arrest. No experimental neuroprotective therapy has translated into clinical benefit. Collaborative efforts to harmonize methods may be essential for translational success. Neurofilament light chain (NfL) is a biomarker of neuroaxonal injury with emerging use in clinical practice, but data on its robustness and association with outcomes in porcine models is limited. Our aim was to use NfL as a quantitative marker to compare brain injury severity in different porcine cardiac arrest models.

**Methods:**

Forty-four pigs (*Sus scrofa domestica*) were resuscitated after cardiac arrest in different experimental models in four laboratories. Plasma samples were collected prior to the arrest and at 4 and 24 h after return of spontaneous circulation. NfL concentration was measured using a Single molecule array (Simoa) immunoassay.

**Results:**

NfL levels changed significantly over time (*p* < 0.001), with similar temporal patterns observed across all laboratories. Inter-laboratory comparisons indicated essentially consistent NfL levels, with modest differences at 24 h. NfL levels at 24 h showed an association with no-flow duration (*n* = 43) in both correlation and univariate regression analyses (*p* = 0.034, *p* = 0.041), while no significant associations were found with functional outcomes (*n* = 26) (*p* = 0.32, *p* = 0.20).

**Conclusion:**

NfL increased consistently across four porcine cardiac arrest models, and higher 24-h NfL levels were associated with longer no-flow duration, supporting its robustness as a biomarker of neuroaxonal injury. Larger studies are needed to clarify its relationship with functional outcome, as the exploratory analyses were underpowered.

## Introduction

Out-of-hospital cardiac arrest (OHCA) remains a major public health issue, with survival rates around 10%.^1^ The majority of deaths following successful cardiopulmonary resuscitation are due to anoxic brain injury.^2^ Several neuroprotective interventions have shown efficacy in experimental settings, but translation to clinical practice has failed. A lack of standardized methodology in experimental cardiac arrest studies may have contributed to this translational failure. Increased and improved collaboration between centers, including comparisons of study designs, severity of insults and outcome measures, is essential for advancing research in the experimental setting.

Biomarkers in peripheral blood are attractive candidates as objective measures of brain injury after cardiac arrest. The Neurofilament light chain (NfL) protein constitutes a subunit of neurofilaments, cylindrical structural proteins located within the cytoplasm of neurons, predominantly in large, myelinated axons.^3^ Clinical findings indicate that NfL levels in peripheral blood are a predictor of functional outcomes as early as 12 h following return of spontaneous circulation (ROSC) in cardiac arrest patients.^4^ A recent clinical study showed superiority in outcome prediction when NfL was compared to other biomarkers of brain injury including Neuron-specific enolase (NSE), Glial fibrillary acidic protein (GFAP) and S100.^5^ Few experimental studies have explored the trajectory of NfL after cardiac arrest and its link to functional outcomes in porcine models.

Our aim was to compare porcine cardiac arrest models in the TRANScontinental Cardiac Arrest Experimental Network for Discovery (TRANSCEND), a collaborative network, using plasma NfL as a quantitative marker of the severity of brain injury in the different models investigated.

## Methods

Four laboratories in Europe and the United States participated in the study: Charles University, Prague, Czech Republic (CU); University of Michigan, Ann Arbor, MI, USA (U-M); Ecole Nationale Vétérinaire d’Alfort (ENVA), Maisons-Alfort, France; and University of Milan, Milan, Italy (UNIMI). The local ethical authorities approved the experiments at all four sites. The experiments were conducted and reported in accordance with EU Directive 2010/63/EU, the 8th edition of the Guide for the Care and Use of Laboratory Animals, the ARRIVE guidelines and Utstein guidelines.^6^, ^7^, ^8^

Forty-four animals (*Sus scrofa domestica*) were resuscitated after cardiac arrest in four experimental models described in Table 1a, Table 1b, Table 2 and Appendix A1. Animals were brought to the laboratories approximately one week prior to the experiment with free access to food and water and were handled with veterinarian support. Demographic, physiologic and experimental data were prospectively collected. Included animals were controls or pilots, and not subjected to any neuroprotective interventions, further background details of the cardiac arrest and resuscitation models are found in publications by Persson et al. (CU),^9^ Wider et al. (U-M),^10^ Daou et al. (ENVA)^11^ and Motta et al. (UNIMI).^12^

**Table 1a:** Animal and cardiac arrest characteristics.

**Variable**	**CU** **(*n* = 11)**	**U-M** **(*n* = 17)**	**ENVA** **(*n* = 7)**	**UNIMI** **(*n* = 9)**	**Total** **(*n* = 44)**
Breed	Landrace × Large white	Yorkshire	Landrace mixture	Italian Landrace	N/A
Arrest type	Induced VF (Pacing RV)	Induced VF (Direct current RV)	Induced VF (Pacing LV)	Ischemic (Ligation LAD)	N/A
Initial rhythm	VF (11/11)	VF (17/17)	VF (7/7)	VF (9/9)	VF (44/44)
Compression type	Mechanical*	Mechanical*	Mechanical*	Mechanical*	N/A
Sex (F/M)	11/0	8/9	7/0	0/9	26/18
Extubation (Y/N)	Yes	No	Yes	Yes	N/A

CU = Charles University, Prague, Czech Republic; U-M = University of Michigan, Ann Arbor, MI, USA; ENVA = Ecole Nationale Vétérinaire d’Alfort, Maisons-Alfort, France; UNIMI = University of Milan, Milan, Italy; N/A = not applicable.

*LUCAS™.

**Table 1b:** Experimental model variables, medians with interquartile range.

**Variable**	**CU** **(*n* = 11)**	**U-M** **(*n* = 17)**	**ENVA** **(*n* = 7)**	**UNIMI** **(*n* = 9)**	**Total** **(*n* = 44)**
No-flow duration (min)	10 (10–10)	10 (5–10)	14 (12–14)	12 (12–13)	10 (10–12)
Interval from cardiac arrest to initial ROSC (min)	16 (15–16)	16 (16–20)	20.5 (17.5–21.5)	18 (17–19)	17 (16–20)
Total compression time (min)	8 (6–8)	10 (6–16)	7 (6–8)	5 (5–7)	8 (6–10)
Total number of shocks (*n*)	4 (3–5)	1 (1–5)	9 (5–11.5)	14 (11–47)	5 (2–10)
Accumulated adrenaline during resuscitation (mg/kg)	0.04 (0.03–0.04)	0.03 (0.03–0.06)	0.03 (0.03–0.03)	0.03 (0.02–0.03)	0.03 (0.03–0.05)
Weight (kg)	59 (56.5–63.5)	53 (50–55)	31 (30.5–32.5)	41 (38–43)	50 (41–56)
Survival time (days)	7 (7–7)	1 (1–1)	1 (1–1.5)	1 (1–4)	1 (1–4)
Interval from cardiac arrest to extubation (h)	32 (31–32.5)	N/A	7 (6–7)	5 (5–5)	7 (5–31)
NDS (%)	0 (0–44)	N/A	84 (64–86)	12.5 (2.5–32.5)	28 (1–64)
Interval from cardiac arrest to NDS (days)	7 (7–7)	N/A	1 (1–1.5)	1 (1–4)	3 (1–7)

CU = Charles University, Prague, Czech Republic; U-M = University of Michigan, Ann Arbor, MI, USA; ENVA = Ecole Nationale Vétérinaire d’Alfort, Maisons-Alfort, France; UNIMI = University of Milan, Milan, Italy; No-flow duratio*n* = interval from start of cardiac arrest to start of compressions; ROSC = return of spontaneous circulation; NDS = percentage of neurological deficit score scale maximum.

Following ROSC, animals were kept on the ventilator (4–33 h) and received intensive care. At three sites (CU, ENVA and UNIMI), the animals were extubated. Planned survival intervals ranged from 1–7 days from cardiac arrest and all animals were euthanized at the end of the experiments or earlier based on criteria for pre-emptive euthanasia.

Plasma EDTA samples were collected at baseline prior to cardiac arrest and at 4 and 24 h after ROSC. Samples were centrifuged and stored at −80°C. Analyses were performed at the Clinical Neurochemistry Laboratory at the University of Gothenburg. Plasma NfL concentration was measured using a Single molecule array (Simoa) immunoassay (Quanterix, Billerica, MA).

Extubated animals were assessed using Neurological deficit scores (NDS) and the final time point measured in each model is reported as percentage of maximum score. The scales used were previously published by Sipos et al.,^13^ Berg et al.^14^ and a modified version of the latter (specified in Appendix A2) at the different institutions (CU, UNIMI, ENVA) respectively. Survival times and functional outcome results are further described in Appendix A1.

A sham group was not part of the harmonized study protocol. To address whether the observed NfL increase could reflect surgical instrumentation, anesthesia or ventilation rather than cardiac arrest, we retrospectively obtained NfL measurements from four sham pigs (ENVA) that underwent anesthesia and instrumentation including 30 min of mechanical ventilation, but no cardiac arrest. Samples from baseline and 6 h were measured with Simoa immunoassay (Quanterix, Billerica, MA) locally at ENVA.

The four laboratories applied different target pressures (systolic pressure ranging from 50 mmHg to 80 mmHg) as well as substantial variations in times to define sustained ROSC (1–20 min). To increase uniformity, the parameter initial ROSC was used in the analyses and was defined as the first time point at which spontaneous circulation reached the pressure threshold.

### Statistics

To evaluate changes in NfL levels over time, non-parametric statistical methods were applied, since the sample sizes were small, and the data violated assumptions of normality according to the Shapiro–Wilk tests and visual inspection of the distributions. All tests were two-tailed, and p-values below 0.05 were considered statistically significant.

To assess temporal changes in NfL levels, a Friedman test was conducted across three measurement points (baseline, 4 h, and 24 h) in the full cohort and per lab. Effect sizes were estimated with Kendall’s W. When significant overall effects were detected, post hoc pairwise Wilcoxon signed-rank tests with Bonferroni correction were performed. Effect sizes in post-hoc comparisons were estimated with rank-biserial correlation (r_rb). To complement these tests, median fold-change values of NfL between baseline and 24 h were calculated to illustrate the magnitude and direction of changes, with 95% confidence intervals estimated using bootstrap resampling with 2000 iterations.

To explore inter-laboratory differences, NfL levels were compared between laboratories at each time point using Kruskal–Wallis tests followed by Bonferroni-corrected pairwise Mann–Whitney U comparisons when appropriate. Effect sizes for the Kruskal-Wallis tests were estimated using epsilon squared (*ε*^2^), while Cliff’s delta (*δ*) was calculated for the post-hoc tests.

Associations between experimental model parameters and NfL levels at the latest measured time point (24 h) were explored. To account for potential non-linear relationships and the limited sample size (total *n* = 26–43), Spearman correlations with bootstrapped confidence intervals were used. Due to the small sample size and the potential for multicollinearity, multivariable regression models were unstable; therefore, each factor was analyzed separately using univariate linear regression, again with bootstrapped confidence intervals. The outcome was log-transformed using the natural logarithm to stabilize variance and allow interpretation of coefficients in relative terms. Shapiro–Wilk tests indicated that residuals were normally distributed in all univariate models. The analyses were explicitly exploratory and aimed to identify potential non-causal associations. Coefficients are reported with 95% confidence intervals, and p-values are provided as indicative rather than confirmatory.

In addition, we performed a simulation to estimate a required sample size for a two-armed study that could detect a 50% reduction in fold-change at 24 h. Median fold-change for each participant was calculated as the ratio of the 24-h measurement to baseline, preserving the paired structure of baseline and 24-h values within individuals. For each simulation, participants were randomly sampled with replacement from the observed data to form a control group. A corresponding treatment group was generated by applying a 50% reduction to each participant’s fold-change, maintaining individual-level variation and the true distribution of the data. Differences between groups were assessed with a two-sided Mann-Whitney U test, and power was defined as the proportion of 2000 simulations per group size yielding *p* < 0.05. Sample sizes from 5 to 30 participants per group were evaluated.

Statistical analyses were performed using R (version 4.5.2; R Core Team, 2025) and RStudio (version 2026.01.0+392; Posit Software, PBC, Boston, MA).

## Results

### Temporal changes

**Fig. 1:**
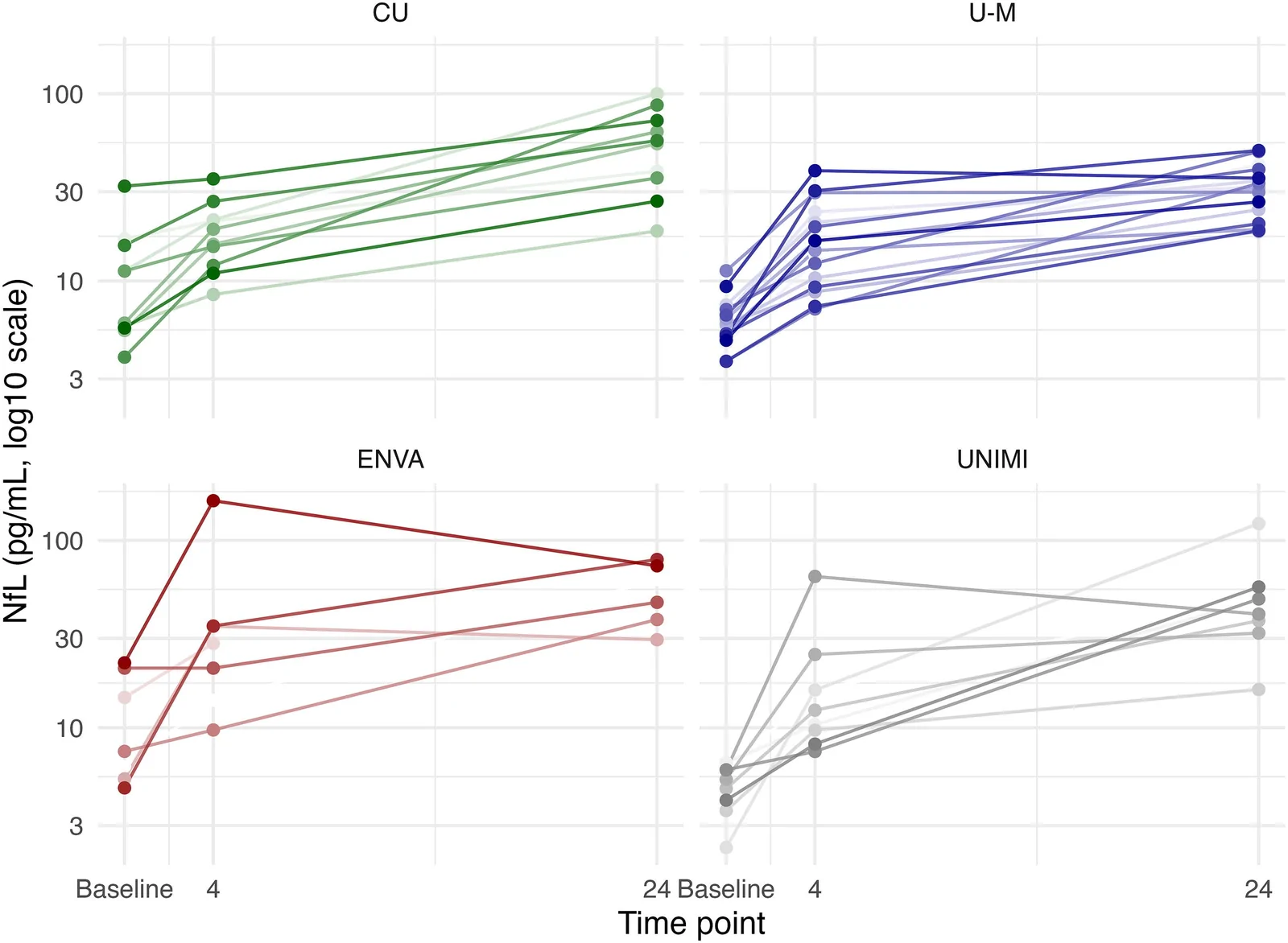
**Trajectory of individual animal Neurofilament light chain (NfL) levels**. CU = Charles University, Prague, Czech Republic; U-M = University of Michigan, Ann Arbor, MI, USA; ENVA = Ecole Nationale Vétérinaire d’Alfort, Maisons-Alfort, France; UNIMI = University of Milan, Milan, Italy.

The results of the NfL measurements are displayed in Table 2 and the trajectories of individual animals are shown in Fig. 1. The Friedman test revealed a significant overall effect of time on NfL levels in the entire cohort and the effect size was large (*p* < 0.001, *W* = 0.89), indicating that NfL concentrations varied significantly across the three measurement points. Post hoc pairwise comparisons confirmed significant differences and large effect sizes; between baseline and 4 h (*p* < 0.001, r_rb = 1), baseline and 24 h (*p* ≤ 0.001, r_rb = 1), and between 4 h and 24 h (*p* ≤ 0.001, r_rb = 0.85). NfL levels thus changed significantly over time in the entire cohort.

**Table 2:** Overview of Neurofilament light (NfL) levels (pg/mL).

**Variable**	**CU**	**U-M**	**ENVA**	**UNIMI**	**Total**
**NfL baseline**					
Numbers	11	17	7	9	44
Missing	0	0	0	0	0
Mean (SD)	11.8 (8.2)	6.2 (1.9)	11.6 (7.5)	4.9 (1.4)	8.2 (5.9)
Median (IQR)	11.3 (5.7–15.7)	5.9 (5.1–7.0)	7.5 (5.8–17.6)	5.3 (4.1–5.9)	6 (5.2–7.9)
min–max	3.9–32.1	3.7–11.3	4.8–22.2	2.3–6.5	2.3–32.1

**NfL 4 h**					
Numbers	11	17	7	9	44
Missing	0	0	0	0	0
Mean (SD)	19.1 (7.8)	17.1 (9.0)	43.6 (53.5)	18.7 (17.8)	22.2 (24.4)
Median (IQR)	18.9 (13.7–22.9)	14.6 (10.4–20.6)	28.2 (17.4–34.8)	12.4 (9.7–15.9)	15.9 (10.8–24.6)
min–max	8.5–35.1	7.1–38.8	9.7–163	7.5–64.2	7.1–163

**NfL 24 h**					
Numbers	11	17	6	9	43
Missing	0	0	1	0	1
Mean (SD)	55 (24.9)	30.8 (10.2)	54.6 (19.8)	50.2 (29.8)	44.4 (23.0)
Median (IQR)	54.2 (37.0–67.3)	31.4 (20.9–35.4)	54.2 (40.0–70.3)	48.2 (37.3–49.8)	38.6 (29.7–54.2)
min–max	18.5–100.0	18.4–49.8	29.5–79.1	16.0–123.0	16.0–123.0

CU = Charles University, Prague, Czech Republic; U-M = University of Michigan, Ann Arbor, MI, USA; ENVA = Ecole Nationale Vétérinaire d’Alfort, Maisons-Alfort, France; UNIMI = University of Milan, Milan, Italy; SD = standard deviation; IQR = interquartile range.

When analyzed separately by laboratory, similar patterns were observed. CU showed a large significant time effect (*p* < 0.001, *W* = 1) with significant pairwise differences across all time points. U-M also demonstrated a robust time effect (*p* < 0.001, *W* = 0.95) with significant differences between all measurement pairs. In ENVA, the overall effect of time was significant (*p* = 0.015, *W* = 0.60), but no post hoc comparisons remained significant after Bonferroni correction, suggesting smaller or unevenly distributed changes. UNIMI exhibited a significant overall time effect (*p* < 0.001, *W* = 0.90), with significant differences between baseline and 4 and 24 h, but not between 4 h and 24 h.

The median fold-change of NfL from baseline to 24 h was 5.06 (95% CI: 4.43–6.99) in the overall cohort. When analyzed by laboratory, the median fold-changes were 3.63 (95% CI: 3.14–9.94), 4.83 (95% CI: 3.79–5.99), 5.30 (95% CI: 2.77–13.2), and 7.89 (95% CI: 6.05–13.8) in CU, U-M, ENVA and UNIMI respectively.

In summary, these results indicate pronounced temporal changes in NfL levels across the cohort, with largely consistent patterns across laboratories, though the magnitude of change varied.

The results from sham animals are presented descriptively in Appendix A3.

### Inter-laboratory comparisons

**Fig. 2:**
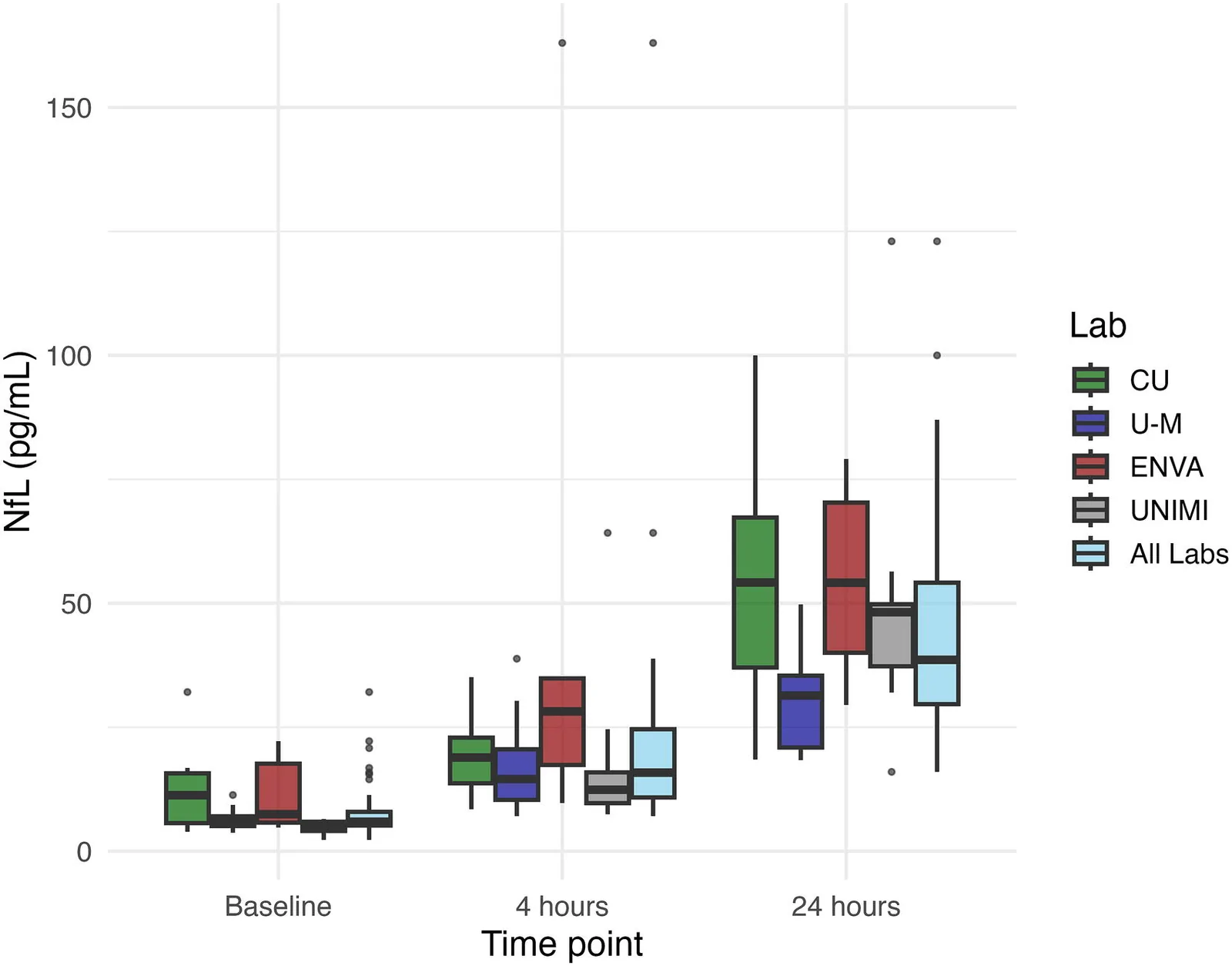
**Boxplots of Neurofilament light chain (NfL) levels**. CU = Charles University, Prague, Czech Republic; U-M = University of Michigan, Ann Arbor, MI, USA; ENVA = Ecole Nationale Vétérinaire d’Alfort, Maisons-Alfort, France; UNIMI = University of Milan, Milan, Italy.

Boxplots for inter-laboratory comparisons are displayed in Fig. 2. At baseline, NfL levels differed significantly between laboratories (*p* = 0.025, *ε*^2^ = 0.16), but no pairwise comparisons reached statistical significance after correction. At 4 h, no significant differences were observed between laboratories (*p* = 0.196, *ε*^2^ = 0.04). At 24 h, a significant overall difference was detected (*p* = 0.0077, *ε*^2^ = 0.23), with post hoc analysis revealing a significant difference between CU and U-M (adjusted *p* = 0.025, *δ* = 0.64), while all other comparisons remained non-significant.

Overall, NfL measurements were largely consistent across laboratories, with only modest inter-laboratory variation observed at the 24-h time point.

### Association analyses

**Fig. 3:**
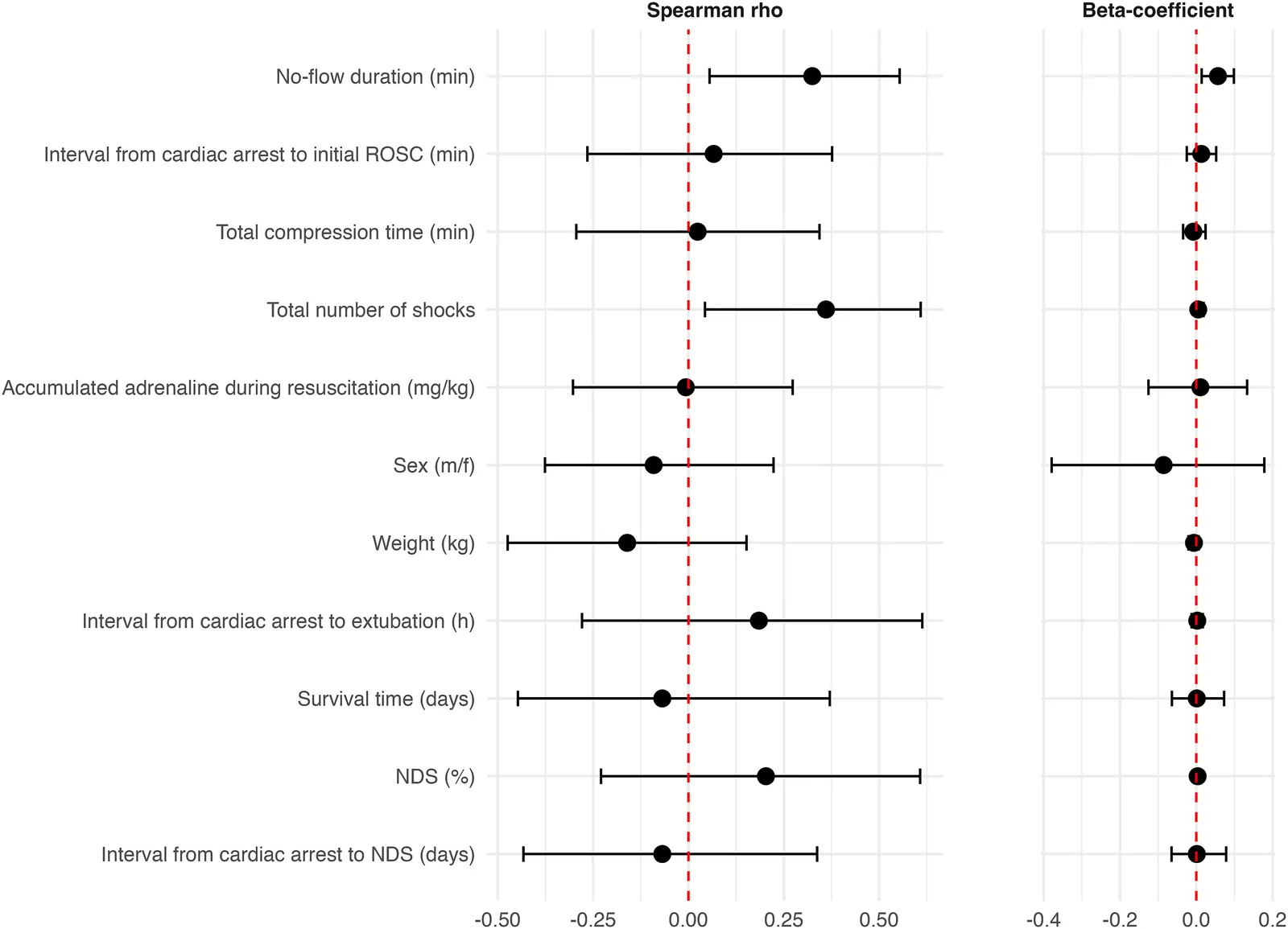
**Overview of Spearman correlations and univariate regressions versus Neurofilament light chain (NfL) levels at 24 h from cardiac arrest, with bootstrapped 95% confidence intervals**.

**Fig. 4:**
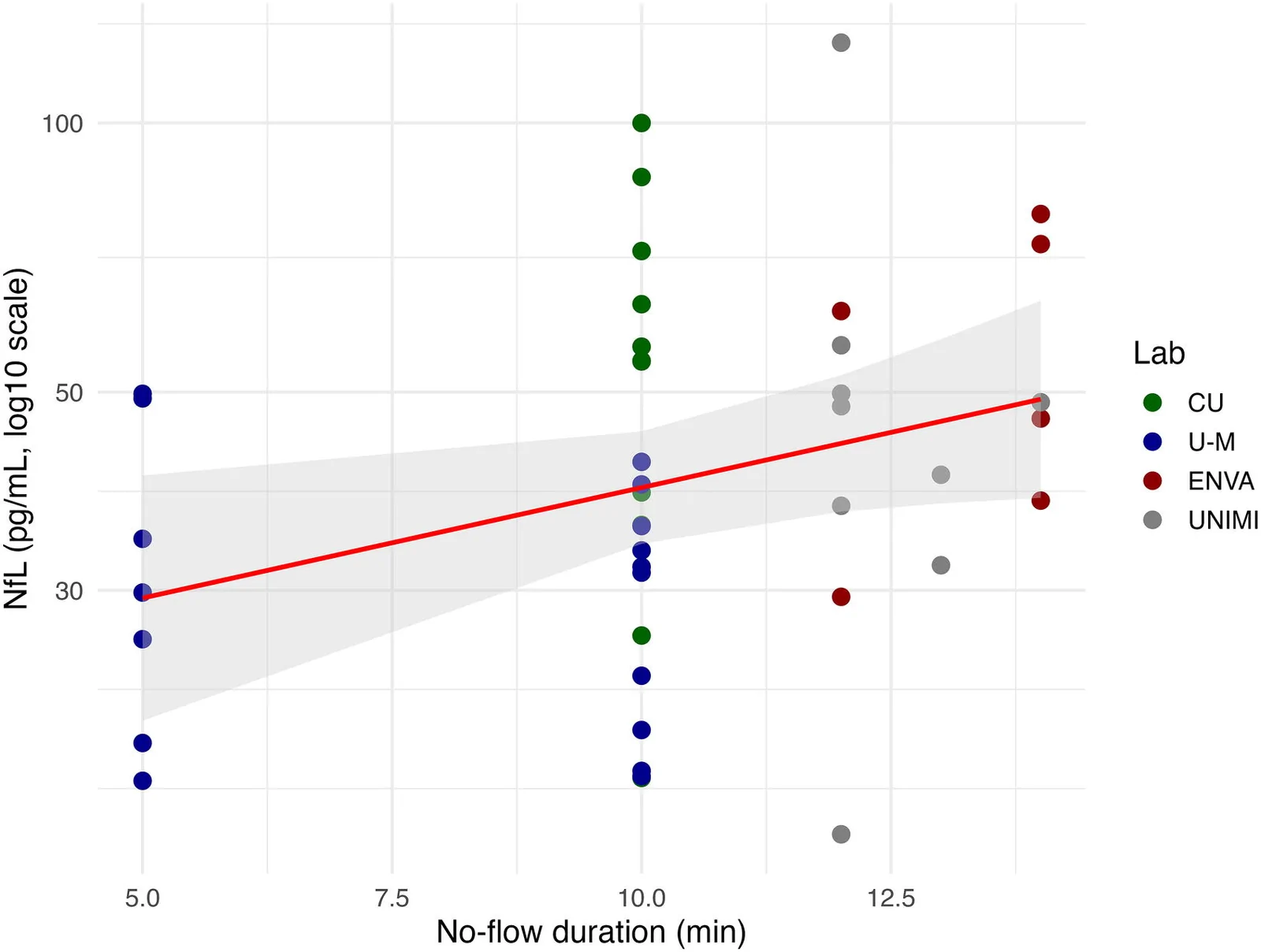
**Scatterplot showing the relationship between Neurofilament light chain (NfL) at 24 h from cardiac arrest and no-flow duration. The solid line represents the linear regression fit, with the shaded area indicating the 95% confidence interval**. CU = Charles University, Prague, Czech Republic; U-M = University of Michigan, Ann Arbor, MI, USA; ENVA = Ecole Nationale Vétérinaire d’Alfort, Maisons-Alfort, France; UNIMI = University of Milan, Milan, Italy.

Spearman correlation tests are presented in Fig. 3. Analyses indicated that both no-flow duration (*p* = 0.034, rho = 0.33) and total number of shocks (*p* = 0.018, rho = 0.36) were positively associated with NfL levels at 24 h. An overview of univariate linear regressions is displayed in Fig. 3. Univariate linear regression showed that the predictor no-flow duration again was significantly positively associated with the biomarker (b = 0.057, *p* = 0.041), explaining 9.8% of the variance in log NfL at 24 h (*R*^2^ = 0.098). Since each factor was analyzed separately without adjustment for other covariates, results should be interpreted with caution. The relationship between no-flow duration and NfL at 24 h is presented graphically in Fig. 4.

No significant association was found between the final functional outcome NDS and NfL at 24 h, in either correlation or regression analyses (*p* = 0.32, *p* = 0.20) respectively (*n* = 26). The relationship between NfL results at 24 h and NDS results are displayed in Appendix A4.

### Simulation-based sample size calculation

Simulations on raw data indicated that to achieve 80% power, the estimated sample size per group should be *n* = 15 to detect a 50% median fold-change in NfL at 24 h. Raw data simulations retained observed variability and skewness, while median fold-change captured effects relative to each baseline. The results of the simulation-based sample size calculation are displayed in Appendix A5.

## Discussion

Findings from this multicenter study demonstrate pronounced temporal changes in NfL levels with patterns largely consistent across all four laboratories. Although the magnitude of change differed between sites, inter-laboratory variation remained modest, underscoring the overall robustness of NfL measurements across the different cardiac arrest models. The observations in this study are generally in line with the preliminary results,^15^ with additional samples and further analyses conducted in the present manuscript.

Few studies have published data on NfL levels and the trajectory after cardiac arrest in swine. NfL levels in the present study are similar to those in publications by Annoni et al., Daou et al., and Vammen et al.^16^, ^17^, ^18^ The study by Vammen et al. with 7 min no-flow duration showed significantly elevated median NfL levels at 48 h after cardiac arrest compared to sham animals (median 63 vs 29 pg/mL), with no clear separation at 24 h. The sampling from CU control animals included in this study continued beyond 24 h and reached peak concentrations at 48 h with similar levels at day 7, as published by Persson et al.^19^

NfL data from our sham animals remained essentially unchanged from baseline to 6 h (median 9 to 12 pg/mL), in contrast to the marked rise already evident at 4 h in resuscitated animals. Although limited by the small number of sham animals, a separate single laboratory batch, a 6-h sampling window only, these data patterns suggest that the temporal increase in NfL is attributable primarily to cardiac arrest and reperfusion injury rather than to anesthesia and instrumentation alone.

The NfL levels in the present study were overall lower than in human cardiac arrest studies using the same immunoassay, especially compared to patients with poor neurological outcome.^4^, ^20^ It should be noted that variations in NfL levels between different assays exist when comparing human samples.^21^ The lower levels in the present study might be due to a less severe brain injury in the experimental models but may also relate to the much smaller brains in relation to body size in swine compared to humans,^22^, ^23^ while the blood volume is similar.^24^, ^25^ This assumption is further supported by supplementary data in Vammen et al. showing median NfL below 300 pg/mL in a subgroup with intracranial pressure consistent with brain death.^18^ The optimal timing for measuring NfL to accurately represent brain injury in swine is unknown. Later time points than those showing predictive properties in humans may be appropriate, which could be due to a greater dilutional effect as described above. Furthermore, previous publications indicate a slow release and a long half-life. Senthil et al. reported increasing NfL levels up to 48 h which stayed elevated 5 days after cardiac arrest.^26^

Longer no-flow duration was consistently associated with higher levels of NfL at 24 h in the present study, which is an important finding. No-flow duration mainly defines the severity of the insult, especially in the experimental setting with short low-flow durations and high-quality CPR.^27^ The effect of no-flow duration may seem limited in our study, but statistical constraints must be considered. Although no-flow duration significantly influenced the neuronal injury biomarker, it accounted for only a small portion of the variance. This limited explanatory power is likely due to the clustered distribution of the ischemic times, with most observations centered around 10 min, the small sample size, and possible biological variability within groups. Regarding low-flow duration, quantified as total compression time in our study, again both the small sample size and the narrow range (interquartile range 6–10 min) may have reduced the power to detect significant associations for a factor with a more modest impact. This must also be acknowledged when considering the lack of a significant association for the interval from cardiac arrest to initial ROSC. It should also be noted that this interval does not account for re-fibrillations or severe hypotensive episodes in the immediate post-ROSC period, which may have affected the ability to detect clear effects. Further, the collection of data yielded discrepancies in the terminology used to define sustained ROSC, although current guidelines for laboratory cardiac arrest research recommend a systolic aortic pressure of 60 mmHg for 10 min to define sustained ROSC.^8^ Future studies could benefit from the adoption of standardized definitions.

Overall, attempts to fit a multivariable linear regression model were hampered by model instability, due to small sample sizes and co-linearity among predictors. For instance, ischemia duration is highly confounded with laboratory as short durations occurred exclusively in one laboratory while longer durations were used by others. As a result, more complex statistical models that attempted to separate the effects of no-flow duration and laboratory became unstable. Consequently, univariate analyses were used to explore potential associations. The results should not be interpreted as causal and need confirmation in larger datasets.

The absence of a significant association between NfL at 24 h and final functional outcome is noteworthy. While the smaller sample size in this cohort (*n* = 26) further reduces statistical power, other factors may also have influenced the results. Timing of NDS assessments varied across centers, and both recovery dynamics and residual sedation may have affected outcomes. An important circumstance is that the NDS scales used do not include any specific cognitive tests and therefore may fail to identify more subtle injuries that could be detected by NfL. Another consideration is that NDS scores may be significantly affected by fatigue and general impairment induced by other organ failures, which are common after cardiac arrest in humans.^28^ The discrepancy between NfL levels and functional outcome has also been observed in other experimental conditions. For instance, plasma NfL levels predict outcome but not cognitive decline in traumatic brain injury models in rats.^29^ There might not be a real “gold standard” for a precise neurological evaluation in animals of cardiac arrest, since precise neurological tests can be used only with moderate dysfunction allowing animal survival. Even in stroke models in rodents, a strong variability is acknowledged.^30^ Moreover, there may be a discrepancy when comparing histology, blood biomarkers and functional outcome in swine, as observed in a recent publication.^19^ These findings underscore the importance of standardized, multimodal outcome assessments after experimental cardiac arrest and the necessity of a prolonged follow-up period.

We also provide essential information on outcome variability and effect size of NfL that can be used to plan future studies. The simulation approach using raw individual-level data generates realistic estimates of required sample size.

### Strengths and limitations

The international multicenter design represents a key strength, increasing the robustness of the findings. Batch analysis of blood samples at one time in one laboratory is also a strength. The retrospective approach and limited sample size constrain the study’s external validity, which should be considered when interpreting the results. A further limitation is the lack of a 24-h sham group, which impairs the ability to assess non-ischemic effects, including those attributable to anesthesia and mechanical ventilation.

## Conclusions

Plasma NfL increased consistently across four independent porcine cardiac arrest models. Higher NfL at 24 h was associated with longer no-flow duration, suggesting that NfL reflects the ischemic insult. Both findings support its robustness as a marker of neuroaxonal injury in this setting. In contrast, NfL at 24 h was not associated with functional outcome; this analysis was exploratory, underpowered, and limited by heterogeneous neurological scoring. NfL therefore shows promise as a standardized injury readout for harmonizing and comparing experimental cardiac arrest models, and our data provide effect-size and sample-size estimates to inform future trials. Whether NfL reflects functionally relevant brain injury requires confirmation in larger studies.

## Availability of data and material

The datasets generated and analyzed during this study are available upon request from the corresponding author.

## Consent for publication

Not applicable.

## CRediT authorship contribution statement

**Olof Persson:** Writing – original draft, Visualization, Project administration, Methodology, Investigation, Formal analysis, Data curation, Conceptualization. **Attila Frigyesi:** Writing – review & editing, Methodology, Formal analysis. **Anna Valerianova:** Writing – review & editing, Investigation. **Mikuláš Mlček:** Writing – review & editing, Investigation. **Renaud Tissier:** Writing – review & editing, Investigation. **Fanny Lidouren:** Writing – review & editing, Investigation. **Matthias Kohlhauer:** Writing – review & editing, Investigation. **Giuseppe Ristagno:** Writing – review & editing, Investigation. **Aurora Magliocca:** Writing – review & editing, Investigation. **Francesca Fumagalli:** Writing – review & editing, Investigation. **Kaj Blennow:** Writing – review & editing, Investigation. **Henrik Zetterberg:** Writing – review & editing, Investigation. **Mohamad Hakam Tiba:** Writing – review & editing, Investigation. **Cindy H. Hsu:** Writing – review & editing, Investigation. **Thomas H. Sanderson:** Writing – review & editing, Investigation. **Robert Neumar:** Writing – review & editing, Project administration, Methodology, Funding acquisition, Conceptualization. **Hans Friberg:** Writing – review & editing, Supervision, Project administration, Methodology, Conceptualization.

## Funding

This work has been funded by Laerdal Foundation Project Support Grant.

## Declaration of competing interest

RT is a stakeholder of a company developing a cooling device (Orixha) for the treatment of the post-cardiac arrest brain injury.

KB has during 2023–2025 served as a consultant, at advisory boards, and has given lectures, produced educational materials and participated in educational programs for AC Immune, ALZPath, AriBio, Beckman-Coulter, BioArctic, Eisai, Lilly, Neurimmune, Novartis, Roche Diagnostics, Sunbird Bio, and Siemens Healthineers; all before Sept 2026, and is since Sept 2026 an employee of Lilly. KB is a co-founder of Brain Biomarker Solutions in Gothenburg AB (BBS), which is a part of the GU Ventures Incubator Program, outside the work presented in this paper.

HZ has served at scientific advisory boards and/or as a consultant for Abbvie, Acumen, Alamar, Alector, Alzinova, ALZpath, Amylyx, Annexon, Apellis, Artery Therapeutics, AZTherapies, Cognito Therapeutics, CogRx, Denali, Eisai, Enigma, LabCorp, Merck Sharp & Dohme, Merry Life, Nervgen, Novo Nordisk, Optoceutics, Passage Bio, Pinteon Therapeutics, Prothena, Quanterix, Red Abbey Labs, reMYND, Roche, Samumed, ScandiBio Therapeutics AB, Siemens Healthineers, Triplet Therapeutics, and Wave, has given lectures sponsored by Alzecure, BioArctic, Biogen, Cellectricon, Fujirebio, LabCorp, Lilly, Novo Nordisk, Oy Medix Biochemica AB, Roche, and WebMD, is a co-founder of Brain Biomarker Solutions in Gothenburg AB (BBS), which is a part of the GU Ventures Incubator Program, and is a shareholder of CERimmune Therapeutics (outside submitted work).

Authors AM and GR serve as Young ERC Editor and Editorial Board Member, respectively, for Resuscitation Plus. They had no role in the editorial assessment, peer-review process, or decision-making concerning this manuscript.

## Appendix A Supplementary material

Supplementary Data 1 Appendix A. Supplementary material comprising Sections A1-A5.
