# Supplementary material for: Neurofilament light chain for assessment of brain injury in four porcine cardiac arrest models
Source: Resusc Plus. 2026 Aug 25;31:101466. doi: 10.1016/j.resplu.2026.101466 (PMC13559832; doi:10.1016/j.resplu.2026.101466)
Supplement: Supplementary file 1 [file mmc1.docx]

Appendix A

Neurofilament Light Chain for assessment of brain injury in four Porcine Cardiac Arrest Models

**A1. Experimental model variabels.**

| **Variable** | **CU** | **U-M** | **ENVA** | **UNIMI** | **Total** |
| --- | --- | --- | --- | --- | --- |
| **No flow duration (min)**  numbers  missing  mean (SD)  median (IQR)  min–max | 11  0  10 (0)  10 (10–10)  10–10 | 17  0  7.9 (2.5)  10 (5–10)  5–10 | 7  0  13.1 (1.1)  14 (12–14)  12–14 | 9  0  12.4 (0.7)  12 (12–13)  12–14 | 44  0  10.2 (2.7)  10 (10–12)  5–14 |
| **Interval from cardiac arrest to initial ROSC (min)**  numbers  missing  mean (SD)  median (IQR)  min–max | 11  0  15.6 (1.7)  16 (15–16)  12–18 | 17  0  17.6 (4.1)  16 (16–20)  10–26 | 7  0  20.2 (3.2)  20.5 (17.5–21.5)  17–26 | 9  0  19.3 (4)  18 (17–19)  17–29 | 44  0  17.9 (3.7)  17 (16–20)  10–29 |
| **Total compression time (min)**  numbers  missing  mean (SD)  median (IQR)  min–max | 11  0  7.2 (1.7)  8 (6–8)  4–10 | 17  0  12.3 (5.8)  10 (6–16)  5–22 | 7  0  8.1 (3.7)  7 (6–8)  5–16 | 9  0  6.3 (2.2)  5 (5–7)  5–10 | 44  0  9.1 (4.8)  8 (6–10)  4–22 |
| **Total number of shocks (n)**  numbers  missing  mean (SD)  median (IQR)  min–max | 11  0  4.5 (2.4)  4 (3–5)  1–10 | 17  0  3.4 (3.6)  1 (1–5)  1–11 | 7  0  8.4 (3.8)  9 (5–11.5)  4–13 | 9  0  29.7 (31.4)  14 (11–47)  2–92 | 44  0  9.8 (17.3)  5 (2–10)  1–92 |
| **Accumulated adrenaline during resuscitation (mg/kg)**  numbers  missing  mean (SD)  median (IQR)  min–max | 11  0  0.04 (0.01)  0.04 (0.03–0.04)  0.02–0.06 | 17  0  0.04 (0.02)  0.03 (0.03–0.06)  0.00–0.06 | 7  0  0.04 (0.02)  0.03 (0.03–0.03)  0.02–0.09 | 9  0  0.03 (0.02)  0.03 (0.02–0.03)  0.02–0.07 | 44  0  0.04 (0.02)  0.03 (0.03–0.05)  0.00–0.09 |
| **Weight (kg)**  numbers  missing  mean (SD)  median (IQR)  min–max | 11  0  60.5 (8.3)  59 (56.5–63.5)  50–75 | 17  0  52.8 (4.4)  53 (50–55)  44–60 | 7  0  32.1 (3.8)  31 (30.5–32.5)  28–40 | 9  0  39.9 (3.8)  41 (38–43)  33–44 | 44  0  48.8 (11.5)  50 (41–56)  28–75 |
| **Survival time (days)**  planned survival  numbers  missing  mean (SD)  median (IQR)  min–max | 7  11  0  6.1 (2)  7 (7–7)  2–7 | 1  17  0  1 (0)  1 (1–1)  1–1 | 1–3  7  0  1.4 (0.8)  1 (1–1.5)  1–3 | 1–4  9  0  2.3 (1.6)  1 (1–4)  1–4 | 1–7  44  0  2.6 (2.4)  1 (1–4)  1–7 |
| **Interval from cardiac arrest to extubation (h)**  numbers  missing  mean (SD)  median (IQR)  min–max | 11  0  31.7 (1)  32 (31–32.5)  30–33 | N/A | 7  0  6.6 (0.6)  7 (6–7)  5.5–7 | 9  0  4.9 (0.6)  5 (5–5)  4–6 | 27  17  16.3 (13.1)  7 (5.2–31)  4–33 |
| **NDS (%)**  numbers  missing  mean (SD)  median (IQR)  min–max | 11  0  23.3 (33.8)  0 (0–44.4)  0–90 | N/A | 7  0  72 (22.4)  84 (64–86)  28–92 | 9  0  21.1 (21.9) 12.5 (2.5–32.5)  0–63.8 | 27  17  35.2 (34.6)  28 (1.2–64)  0–92 |
| **Interval from cardiac arrest to NDS (days)**  numbers  missing  mean (SD)  median (IQR)  min–max | 11  0  6.1 (2)  7 (7–7)  2–7 | N/A | 7  0  1.4 (0.8)  1 (1–1.5)  1–3 | 9  0  2.3 (1.6)  1 (1–4)  1–4 | 27  17  3.6 (2.6)  3 (1–7)  1–7 |

CU = Charles University, Prague, Czech Republic; U-M = University of Michigan, Ann Arbor, MI, USA; ENVA = Ecole Nationale Vétérinaire d’Alfort, Maisons-Alfort, France; UNIMI = University of Milan, Milan, Italy. N/A = not applicable; No-flow duration = interval from start of cardiac arrest to start of compressions; ROSC = return of spontaneous circulation; NDS = percentage of neurological deficit score scale maximum.

**A2. Neurological deficit score (NDS) used by Ecole Nationale Vétérinaire d’Alfort, Maisons-Alfort, France (ENVA).**

| **Signs Description** | | | |
| --- | --- | --- | --- |
| **Appetite** | Total food consumed | 0 |  |
|  | More than 50% of the food consumed | 1 |  |
|  | 50% of the food consumed | 2 |  |
|  | Less than 50% of the food consumed | 3 |  |
|  | No food eaten | 4 |  |
| **Standing position** | Normal standing position | 0 |  |
|  | Unstable when standing | 1 |  |
|  | Standing position with support (leans against the wall) | 2 |  |
|  | Rises in response to stimulation | 3 |  |
|  | Lying down, not responding to stimulation | 4 |  |
|  | Motionless | 5 |  |
| **Head position** | Head up | 0 |  |
|  | Head raised in response to stimulation | 1 |  |
|  | Unable to lift head | 2 |  |
| **Vocalization** | Normal and spontaneous vocalization | 0 |  |
|  | Vocalization in response to stimulation | 1 |  |
|  | No vocalization | 2 |  |
| **Movement** | Walk with vitality | 0 |  |
|  | Spontaneously unstable walking | 1 |  |
|  | Unsteady walking in response to stimulation | 2 |  |
|  | Unable to walk | 3 |  |
| **Motor function of forelimbs** | No motor problems | 0 |  |
|  | Slight weakness (instability during movement) | 1 |  |
|  | Moderate paresis (instability when standing) | 2 |  |
|  | Severe paresis | 3 |  |
|  | Complete paralysis | 4 |  |
| **Motor function of hind limbs** | No motor problems | 0 |  |
|  | Slight weakness (instability during movement) | 1 |  |
|  | Moderate paresis (instability when standing) | 2 |  |
|  | Severe paresis | 3 |  |
|  | Complete paralysis | 4 |  |
| **Face** | Symmetrical Face (during rest and movement) | 0 |  |
|  | Facial paralysis | 1 |  |
| **Total** | **Maximum score** | **25** |  |
|  | **Total over 100%** | **100** |  |

0%, animal is in a good neurological condition; 100%, brain death

**A3. Boxplot of Neurofilament light chain (NfL) levels including sham animals.**

**Figure A3.** Boxplots of Neurofilament light chain (NfL) levels. CU = Charles University, Prague, Czech Republic; U-M = University of Michigan, Ann Arbor, MI, USA; ENVA = Ecole Nationale Vétérinaire d’Alfort, Maisons-Alfort, France; UNIMI = University of Milan, Milan, Italy, Sham = 6 h Sham samples.

**Table A3. Sham animal NfL results (pg/mL)**

| **Variable** | **Sham** |
| --- | --- |
| **NfL Baseline**  numbers  missing  mean (SD)  median (IQR)  min–max | 4  0  9.8 (4.4)  8.8 (7.2–11.3)  5.6–15.9 |
| **NfL 6 h**  numbers  missing  mean (SD)  median (IQR)  min–max | 4  0  12.1 (3.3)  11.8 (10.2–13.8)  8.5–16.3 |

**A4. Scatterplot showing the relationship between Neurofilament light chain (NfL) at 24 hours from cardiac arrest and Neurological deficit score (NDS).**

NfL = Neurofilament light chain at 24 hours; NDS = Neurological deficit score, as percentage of maximum score. CU = Charles University, Prague, Czech Republic; ENVA = Ecole Nationale Vétérinaire d’Alfort, Maisons-Alfort, France; UNIMI = University of Milan, Milan, Italy.

**A5. Power curve for detecting a 50% median fold-change in Neurofilament light chain (NfL) levels at 24 hours after cardiac arrest in a two-armed study.**
